# Cholera Epidemica; or Indo-Russian Cholera Morbus

**Published:** 1831

**Authors:** 


### Cholera Epidemica; or Indo-Russian Cholera Morbus.

We have been preparing an extended article on this wide-wasting pestilence, and have brought it down to the time of writing this short notice. But we find that no limit is yet put to the ravages of the disease—no satisfactory history of its European progress compiled—no probable *cause* of the malady ascertained—and no consolatory prospects of a successful treatment brought to light! Such, at least, is the case at the moment we now write, viz. the middle of August. Under these circumstances, we have thought it best to wait till the close of the year, when, in all human probability, the epidemic will have spent its force (perhaps paid us a visit)—and our boards of health and commissioners of investigation will have had time to arrange their information, and lay it before the public at large. The successive, and, we are sorry to say, the *successful* campaigns, which this enemy of the human race has hitherto made in his march through the wide regions of the East, and now towards the shores of the Atlantic, render it probable that, if he does approach our coast in some shape or other, and with more or less of his exterminating virulence—this will not be the year, even if he have added to his original destructive and gigantic powers the terrible auxiliary CONTAGION. But, whether the disease be epidemic, contagious, or both united, it will be prudent, during the ensuing Winter's cessation of the conflict, to collect all our information, and array, if possible, all our resources of science and art—of hygiene and therapœia, preparatory to the Summer of 1832.

The mortality and the extensive prevalence of cholera at St. Petersburg, where our medical commissioners are now in active observation, will furnish them with ample opportunities of investigating the effects of remedies, the post-mortem changes, and the nature of the disease, more especially as to its contagious or non-contagious character. Our wishes and our hopes coincide with our expectations, that this dreadful malady will be found to possess but a low range of contagious power, such as arises *contingently* in various epidemic diseases, not originally endued with that character. Should it be otherwise—should cholera prove half so infectious as the fears and the preconceptions of people would appear to determine, we tremble for the safety of our countrymen, who are now in daily and hourly contact with this NOVA PESTIS. Considering, indeed, that the commissioners are subjected to the same primary causes of cholera which are striking down thousands around them—that they are unavoidably exposed to contagion (if it exists) in all its shades of force and concentration—that their minds are kept in a state of incessant anxiety, doubt, and, we may add, dismay—that their bodies must be worn down with toil of the most insalutary kind—considering these and many other circumstances, we say it will be almost a miracle if they escape. If their situation be viewed with a philosophic and an unprejudiced eye, an *attack* of the disease would afford no proof of contagion—though an *escape* would offer strong presumptive evidence on the other side of the question. Be this as it may, the number of medical observers now on the theatre of the epidemic, from France, England, and Germany, will furnish us, before the conclusion of the year, with ample materials for reflection, though we fear that these materials will tend little to unanimity of sentiment on either side of the Channel. The doctrine of contagion will be adopted and opposed with all the zeal of enthusiastic conviction, while almost every individual will maintain that his own mode of treatment is the best. If the present time, therefore, be one of doubt and uncertainty, the future, should the epidemic invade us, will be one of distraction and alarm. We have often been asked what we ourselves would do, in the event of meeting with a case of the malignant cholera? Our opinion may not be worth publicity; but, as it contains that which we would wish to be pursued in our own case, it has at least the merit of sincerity.

The first step which we would advise would be, the placing the patient in a warm bed, where he is to be chafed with hot flannels all over the body, while a hot bath is preparing. The flannels might be impregnated with ardent spirits; and hot water, as soon as procured, substituted for the dry flannel. Mean time, we would administer three grains of opium and ten of calomel instanter, followed by moderate potations of warm brandy and water, while the skin was cold, and the circulation concentrated about the internal organs. We would be inclined to repeat three grains of calomel and one of opium every two or three hours, till re-action took place, and the tide of the circulation flowed to the surface. As soon as the bath was ready, we would place the patient in it, having put a good allowance of salt and mustard in the water. If, by these means, heat be restored on the skin, and the spasms and vomiting be allayed, the great danger is over, and the management of the febrile re-action requires but very moderate skill. The injection of 50 or 100 drops of Batley's sedative, or common laudanum, in a small quantity of starch or gruel, into the rectum, might be beneficial. These, we conceive, are the main indications of treatment; and if they fail, we apprehend that most of the complicated machinery of therapeutics recommended, more from speculation than experience, will be found to share the same fate. Towards the close of the Periscope, we may probably have something to communicate respecting the treatment pursued by our brethren in Russia.

